# Sense of Mastery Explains Social Patterning of Health

**DOI:** 10.3390/healthcare13131511

**Published:** 2025-06-24

**Authors:** Shervin Assari, Babak Najand, Alexandra Donovan

**Affiliations:** 1 Department of Internal Medicine, Charles R Drew University of Medicine and Science, Los Angeles, CA 90059, USA; alexandradonovan@cdrewu.edu; 2 Department of Psychiatry, Charles R Drew University of Medicine and Science, Los Angeles, CA 90059, USA; 3 Department of Family Medicine, Charles R Drew University of Medicine and Science, Los Angeles, CA 90059, USA; 4 Department of Urban Public Health, Charles R Drew University of Medicine and Science, Los Angeles, CA 90059, USA; 5 Marginalization Related Diminished Returns Center, Los Angeles, CA 90059, USA; najand.babak@gmail.com

**Keywords:** cross-national study, education, employment, financial insecurity, health behaviors, mediation, perceived control, sense of mastery, social determinants of health, socioeconomic status, structural equation modeling, well-being

## Abstract

Background: Social determinants of health—including both adversity and socioeconomic position—are known to shape physical health, health-related behaviors, and overall well-being. However, the psychological mechanisms that link these determinants to diverse outcomes remain insufficiently explored across international contexts. Objective: The objective of this study is to test whether sense of mastery and control over one’s life mediates the associations between key stressors (childhood abuse, financial insecurity) and socioeconomic resources (education, employment, and marital status) with a wide range of outcomes spanning health, behaviors, and well-being. Methods: Using cross-sectional data from Wave 1 of the Global Flourishing Study (GFS), we analyzed responses from more than 200,000 adults in 23 countries. Predictors included exposure to childhood abuse, perceived financial insecurity, and indicators of socioeconomic position (education, employment, and marital status). Outcomes included self-rated physical and mental health, depression, anxiety, smoking, drinking, physical activity, life satisfaction, and happiness. Structural equation modeling (SEM) was used to evaluate both direct and indirect (mediated) effects through sense of mastery and control over life. Results: Stressors were associated with poorer health, higher engagement in risk behaviors, and lower well-being. In contrast, higher levels of education, employment, and being married were linked to more favorable outcomes. In all tested models, sense of mastery and control over life significantly mediated the effects of both stressors and socioeconomic resources on health, behaviors, and well-being outcomes. Conclusions: Sense of mastery and control over life may represent key psychological pathways linking both adversity and social advantage to diverse health-related outcomes. Interventions that enhance individuals’ perceived control may offer cross-cutting benefits to improve health, promote well-being, and reduce behavioral risk factors globally.

## 1. Introduction

Social determinants of health (SDOH), encompassing both adverse life experiences and socioeconomic resources, play a central role in shaping individual and population-level differences in health, behaviors, and subjective well-being [1]. Adverse exposures such as childhood abuse and ongoing financial insecurity have been widely associated with poorer health outcomes, increased psychological distress, and heightened engagement in health-compromising behaviors [2]. Conversely, higher levels of education, stable employment, and supportive social relationships—often measured through marital status—are linked to better physical and mental health, lower rates of unhealthy behaviors such as smoking and drinking, and greater life satisfaction and happiness [3]. Yet, the mechanisms by which these upstream social forces translate into such diverse individual outcomes remain an area of ongoing inquiry [4].

While the associations between social determinants and health-related outcomes are well established, fewer studies have explored the psychological processes that might explain how these social factors “get under the skin” [5,6]. Much of the existing research has focused on either specific stressors (e.g., childhood trauma) [7,8] or specific resources (e.g., education) [9,10], and often examined them in isolation rather than simultaneously. Even fewer studies have investigated whether a single psychological factor might serve as a common pathway linking both adverse and protective social experiences to a wide range of outcomes—including not just health, but also behavior and broader well-being [11,12].

A promising construct in this regard is sense of mastery, or perceived control over one’s life. Although the terms *sense of mastery*, *control*, and *perceived control* have slight conceptual differences, they broadly refer to individuals’ belief in their ability to influence life outcomes and navigate challenges. This perceived agency represents a core psychological resource that shapes how individuals respond to their social environment [4]. The concept is grounded in theoretical models such as the stress process framework, which posits that individuals with a stronger sense of control are more resilient in the face of stress and better able to mobilize coping resources [13,14]. A growing body of research has linked sense of mastery and control to improved physical and mental health, reduced substance use, greater life satisfaction, and enhanced psychological functioning. However, the role of sense of mastery as a potential mediator of the effects of both social adversity and advantage across multiple outcome domains has not been fully tested—particularly in global, culturally diverse populations.

Multiple theories suggest that sense of mastery, control over one’s life, and perceived control are essential drivers of human behavior and central to achieving and maintaining good health. For instance, Social Cognitive Theory [15] emphasizes the role of self-efficacy—the belief in one’s ability to influence events—as a critical factor shaping behavior and outcomes. Locus of Control Theory [16] distinguishes between individuals who perceive outcomes as contingent on their own actions (internal locus) versus those who see them as determined by external forces, with internal locus consistently linked to better health behaviors. The Theory of Planned Behavior [17,18] also highlights perceived behavioral control as a key predictor of intention and action. In addition, the stress process model [19] positions mastery as a psychosocial resource that buffers the negative effects of stressors on mental health, especially in the face of chronic adversity. These frameworks converge on the idea that individuals who feel a greater sense of agency are more likely to engage in proactive health behaviors and cope more effectively with life’s challenges, ultimately promoting physical and mental well-being.

### Aims

Given these gaps, the current study aims to examine whether sense of mastery and control over life mediates the associations between both negative (childhood abuse, financial insecurity) and positive (education, employment, marital status) social determinants and a comprehensive set of outcomes that include physical and mental health, health-related behaviors, and subjective well-being. In a multi-national study [20,21,22], we test whether sense of mastery serves as a consistent psychological bridge linking social conditions to individual functioning across these three domains. We hypothesize that sense of mastery will mediate the effects of both adversity and advantage, suggesting that perceived control may be a key pathway through which social environments shape human flourishing.

## 2. Methods

### 2.1. Study Design and Data Source

This study utilized cross-sectional data from Wave 1 of the Global Flourishing Study (GFS) [20,21,22], a large-scale, international research initiative designed to examine the determinants and distribution of human flourishing across culturally and geographically diverse populations. For this report, we completed and followed the STROBE guideline/checklist for cross-sectional studies [23,24].

### 2.2. Data Source

The GFS is a longitudinal panel study conducted by researchers from Harvard University, Baylor University, Gallup, and the Center for Open Science. Wave 1 was fielded in 2022 and included over 200,000 adults aged 18 and older from 23 countries. The following countries were selected to ensure global diversity in socioeconomic, cultural, and political contexts: Argentina, Australia, Brazil, Egypt, Germany, Hong Kong, China, India, Indonesia, Israel, Japan, Kenya, Mexico, Nigeria, the Philippines, Poland, South Africa, Spain, Sweden, Tanzania, Türkiye, the United Kingdom, and the United States.

Sampling within each country aimed for national representativeness with respect to age, sex, and region, using stratified and quota-based sampling frameworks implemented through Gallup’s international survey infrastructure. Data collection was carried out using standardized self-administered online or phone-based survey instruments. All respondents provided informed consent prior to participation. The GFS received ethical approval from the Institutional Review Board at Harvard University. The present analysis used de-identified, publicly available data and was therefore exempt from additional human subjects review [20,21,22].

### 2.3. Analytical Sample

The analytical sample included all adult participants drawn from 23 countries. The inclusion criteria required participants to have at least partial data on key Wave 1 variables, particularly rurality, educational attainment, and at least one of the outcome measures. Participants with valid data on any of the outcomes were retained in the analysis, as structural equation modeling (SEM) does not require complete data and employs full information maximum likelihood (FIML) [25,26] estimation to handle missingness. The final sample size was 207,919.

## 3. Measures

### 3.1. Predictors: Social Determinants

Two categories of social determinants were examined as predictors: stressors and socioeconomic resources.

Childhood Abuse. Childhood abuse was assessed with a single binary item asking respondents whether they experienced abuse during childhood (0 = No, 1 = Yes) [27].

Perceived Financial Insecurity. Perceived financial insecurity was measured through a single item assessing how financially stable participants currently feel. Higher scores reflected greater financial insecurity and a lower perceived sense of control over finances.

Socioeconomic resources were measured using conventional demographic indicators:

Educational Attainment. Education was recorded as a categorical variable representing the highest level of formal education and recoded into three levels: low, middle, and high education.

Unemployment. Unemployment status was dichotomized (1 = unemployed; 0 = employed, student, retired, or not in the labor force).

Marital Status. Marital status was dichotomized (1 = married or cohabiting; 0 = single, divorced, separated, or widowed).

### 3.2. Mediator

Sense of Mastery. Sense of mastery, also called control over one’s life or perceived control, was assessed using a single-item measure where respondents rated their agreement with a statement reflecting perceived agency and control (e.g., “I feel I am in control of my life”). Responses were coded such that higher values indicated greater sense of mastery.

To ensure clarity and consistency, we refer to our mediating variable throughout this manuscript as “sense of mastery.” Although measured using a single item, we believe this item captures the core psychological construct of perceived mastery—that is, an individual’s belief in their ability to influence events and outcomes in their life. While closely related to constructs such as sense of control or perceived control, we intentionally use sense of mastery as our central term. Given the conceptual overlap among these constructs, particularly in large-scale cross-national studies, we consider our measure to represent a general sense of mastery/control over one’s life and use it as a mediator in our models accordingly.

### 3.3. Outcomes

We examined a broad set of outcomes categorized into three domains: health, behaviors, and well-being.

### 3.4. Health Outcomes

Self-Rated Physical Health. Self-rated physical health was assessed using a single item rated on a 0–10 scale, with higher scores indicating better physical health.

Self-Rated Mental Health. Self-rated mental health was similarly assessed on a 0–10 scale, with higher scores reflecting better mental well-being.

Depression Symptoms. Depression symptoms were measured using a single-item indicator assessing frequency of depressive affect.

Anxiety Symptoms. Anxiety symptoms were measured using a brief two-item scale assessing frequency of worry and anxiety.

### 3.5. Behavioral Outcomes

Cigarette Smoking. Smoking status was coded as a binary variable (1 = current smoker; 0 = non-smoker), based on smoking behavior in the past 7 days.

Alcohol Drinking. Drinking alcohol was also coded as a binary variable (1 = currently drinks alcohol; 0 = does not), based on behavior in the past 7 days.

Physical Activity. Physical activity (exercise) was measured by frequency of engagement in exercise or movement, reported on a standardized response scale.

### 3.6. Well-Being Outcomes

Life Satisfaction. Life satisfaction was assessed using a single-item scale rated from 0 to 10, with higher scores indicating greater satisfaction with life overall.

Happiness. Sense of happiness was also measured using a single-item scale from 0 to 10, with higher scores indicating greater experienced happiness.

### 3.7. Statistical Analysis

We employed structural equation modeling (SEM) to assess whether sense of mastery and control over one’s life mediated the relationship between social determinants (stressors and socioeconomic resources) and outcomes across three domains: health, behaviors, and well-being. SEM was selected for its capacity to simultaneously estimate complex mediation pathways, incorporate multiple dependent variables, and account for measurement error. All models were estimated using maximum likelihood estimation with robust standard errors. Indirect effects through sense of mastery were tested using nonparametric bootstrapping with 5000 replications to generate bias-corrected 95% confidence intervals. We reported standardized path coefficients (β) for all direct and indirect associations.

**Figure 1:**
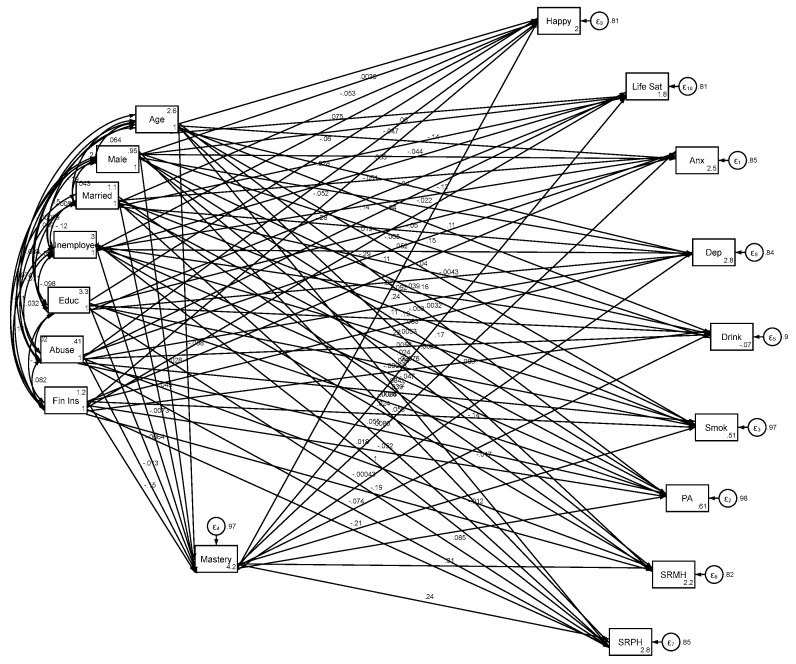
Summary of SEM on association between social determinants and health and well-being.

The structural equation model (SEM) depicted in Figure 1 presents a mediation framework designed to test whether sense of mastery and control over one’s life mediates the relationships between social determinants and a broad array of health, behavioral, and well-being outcomes. On the left side of the model are the exogenous predictor variables, which represent the core social determinants. These include two stressors—childhood abuse and perceived financial insecurity—and three socioeconomic resources—educational attainment, employment status, and marital status. These variables are theorized to influence outcomes both directly and indirectly via their association with the mediator.

At the center of the model is the mediator, sense of mastery and control over life, which is conceptualized as a psychological construct capturing perceived autonomy, agency, and self-determination. This observed variable is hypothesized to be influenced by the five predictor variables and, in turn, to predict a diverse set of outcome variables.

On the right side of the diagram are the endogenous outcome variables, grouped into three broad domains. The health domain includes self-rated physical health, self-rated mental health, depressive symptoms, and anxiety symptoms. The behavioral domain includes smoking, drinking, and physical activity. The well-being domain includes life satisfaction and happiness. Arrows from the social determinants to these outcomes represent direct effects, while arrows passing through the sense of mastery construct represent indirect, mediated effects.

The diagram visually illustrates a full mediation model, in which all social determinants are allowed to influence all outcome variables both directly and through sense of mastery. The structural paths from predictors to the mediator and from the mediator to each outcome reflect hypothesized causal pathways, while covariances were included among predictors. This SEM framework enables simultaneous testing of complex mediation across multiple dependent variables, providing insight into the extent to which psychological perceptions of control serve as a bridge between social conditions and individual functioning. Another strength of structural equation modeling (SEM) is its ability to include multiple outcome variables within a single model. In our case, we simultaneously analyzed nine outcomes, which offers a clear advantage over models that handle each outcome separately. This approach allows a single SEM to estimate numerous total, direct, and indirect effects, while conserving degrees of freedom and reducing the likelihood of false positives by avoiding multiple separate tests.

## 4. Results

### 4.1. Participants

Table 1 displays the geographic distribution of the 207,919 participants included in the analytical sample, spanning 23 countries. The United States contributed the largest share, making up 18.43% of the total sample, followed by Japan at 9.88% and Sweden at 7.25%. Other countries with substantial representation included Brazil (6.35%), India (6.14%), Kenya (5.48%), and Poland (5.00%). Countries with smaller sample shares included Türkiye (0.71%), South Africa (1.28%), and Hong Kong (1.45%). The overall sample reflects broad global coverage, incorporating participants from regions across North and South America, Europe, Asia, and Africa.

Table 2 presents the results of the structural equation model (SEM) testing whether sense of mastery and control over life mediates the associations between social determinants and multiple outcomes across health, behaviors, and well-being (Figure 1).

### 4.2. Predictors of Sense of Mastery

Several social determinants were significantly associated with sense of mastery. Financial insecurity emerged as the strongest negative predictor (*β* = −0.152, *p* < 0.001), followed by older age (*β* = −0.058, *p* < 0.001) and being unemployed (*β* = −0.007, *p* = 0.001). Experiencing childhood abuse (ACE) also predicted lower sense of mastery (*β* = −0.013, *p* < 0.001). Conversely, being male (*β* = 0.028, *p* < 0.001) and being married (*β* = 0.045, *p* < 0.001) were associated with higher sense of mastery. Educational attainment was weakly but significantly negatively associated with sense of mastery (*β* = −0.006, *p* = 0.004).

### 4.3. Sense of Mastery as a Mediator: Health Outcomes

Sense of mastery was significantly and inversely associated with both anxiety (*β* = −0.089, *p* < 0.001) and depression (*β* = −0.143, *p* < 0.001), and positively associated with self-rated physical health (*β* = 0.243, *p* < 0.001) and self-rated mental health (*β* = 0.309, *p* < 0.001). These associations suggest that individuals with a stronger sense of control reported better overall health and fewer mental health symptoms.

### 4.4. Sense of Mastery as a Mediator: Well-Being Outcomes

Mastery was also positively associated with greater happiness (*β* = 0.240, *p* < 0.001) and life satisfaction (*β* = 0.175, *p* < 0.001). These findings reinforce the role of perceived control as a key determinant of psychological well-being in global samples.

### 4.5. Sense of Mastery as a Mediator: Behavioral Outcomes

In the behavioral domain, sense of mastery was positively associated with higher physical activity (*β* = 0.085, *p* < 0.001), and inversely associated with smoking (*β* = −0.012, *p* < 0.001) and drinking (*β* = −0.017, *p* < 0.001). Although smaller in magnitude, these associations indicate that individuals with higher perceived control were less likely to engage in substance-related risk behaviors and more likely to maintain active lifestyles.

Overall, these findings support the hypothesis that sense of mastery and control over one’s life is a robust and consistent psychological mediator of the effects of social determinants on multiple aspects of health, behavior, and well-being.

## 5. Discussion

This study investigated whether sense of mastery and control over one’s life mediates the relationship between multiple social determinants—including both stressors and socioeconomic resources—and outcomes related to health, behavior, and well-being. Using data from a large and culturally diverse international sample, we found consistent evidence that sense of mastery explained the effects of both negative exposures (childhood abuse, financial insecurity) and positive social resources (education, employment, marital status) on all tested outcomes. This pattern held across physical and mental health, risk-related behaviors, and indicators of subjective well-being, such as life satisfaction and happiness.

These findings align with theoretical models that view sense of control as a core psychological process through which social structures affect individual outcomes [28,29]. The stress process model [30,31], for instance, conceptualizes sense of mastery as a resource that enables individuals to interpret life stressors as manageable and to respond in adaptive ways. Our results extend this framework by showing that sense of mastery is not only protective in the face of adversity but also appears to explain how social resources such as education and employment exert their beneficial effects. In other words, individuals with stable employment may feel more in control of their lives, and this higher sense of control, in turn, promotes healthier behaviors, better health, and greater psychological well-being [32,33].

These findings have theory, research, and practice implications. Theoretically, our results suggest that sense of mastery may be a multicultural, transdiagnostic, and cross-domain mediator, helping to unify diverse strands of research on social determinants, health behaviors, and psychological functioning. Practically, these findings point to sense of mastery and perceived control as potential intervention targets relevant to multiple countries. Programs that aim to improve individuals’ sense of control—through skill-building, empowerment strategies, or improved access to information and resources—may not only reduce the harmful effects of adversity but also amplify the benefits of socioeconomic advantage [34,35,36]. For example, public health initiatives or clinical interventions that incorporate sense of mastery-enhancing components may have cross-cutting benefits across multiple outcomes.

Our pooled analysis revealed a somewhat counter-intuitive association between higher educational attainment and lower levels of sense of mastery. This unexpected finding that higher educational attainment was slightly negatively associated with perceived mastery (*β* = −0.006, *p* = 0.004) warrants careful consideration. We interpret this cautiously given the multi-national nature of the sample. The psychological and social meaning of education may differ across cultural contexts, potentially shaping its relationship with constructs like mastery in distinct ways. While our focus was on identifying general patterns across countries, we acknowledge that country-specific dynamics—such as variations in labor markets, educational quality, or social mobility—may influence how education relates to perceived control over life. Future research would benefit from disaggregating these associations at the national level to examine whether and how the education–mastery link varies by country, which could illuminate culturally specific mechanisms that are not visible in pooled analyses.

While this association is small, it challenges the common assumption that education universally enhances one’s sense of control. One possible explanation is that in certain cultural or socioeconomic contexts, higher education may increase awareness of structural barriers, social inequalities, or unattainable expectations—particularly in low-resource settings or among marginalized groups—ultimately diminishing feelings of personal control. Additionally, the value and outcomes of education can vary widely across countries; in some settings, higher education may not translate into better employment or financial stability, thereby limiting its impact on mastery. These findings highlight the importance of examining how the meaning and consequences of education are shaped by broader social and cultural environments.

In this study, we modeled a single mediating variable—sense of mastery—while examining multiple indicators of socioeconomic position (SEP), including educational attainment, employment status, and marital status, as well as a wide array of health and well-being outcomes such as self-rated physical and mental health, depression, anxiety, smoking, drinking, physical activity, life satisfaction, and happiness. While structural equation modeling (SEM) allowed us to examine both direct and indirect effects of SEP on these outcomes through sense of mastery, our analytic framework imposed certain constraints on interpretability. Specifically, due to the interrelated nature of the SEP indicators, we intentionally refrained from quantifying the percentage of mediation or the proportion of variance explained by mastery. This decision was rooted in the recognition that education, employment, and marital status do not operate independently; rather, they are deeply intertwined and often influence each other in complex, bidirectional ways. For example, education can affect health not only directly but also indirectly through increasing employment opportunities and the likelihood of stable marital relationships—both of which are independently associated with health and well-being. In such a context, estimating the proportion of the effects mediated by a cognitive construct like sense of mastery, while ignoring the overlapping pathways among the SEP indicators, would risk oversimplifying the underlying mechanisms. We believe that attempting to isolate the mediation effect of sense of mastery without acknowledging these interdependencies would result in potentially misleading conclusions. Therefore, our goal was not to partition effects into neat percentages, but rather to demonstrate that sense of mastery is one plausible psychological pathway linking SEP to multiple health-related outcomes, amid a broader and more entangled network of structural and interpersonal processes.

The Global Flourishing Study (GFS) is a newly released dataset, and while it offers unprecedented opportunities for cross-national research on health and well-being, many foundational questions remain unanswered. To date, limited research has examined whether the relationships among key variables—such as socioeconomic position, psychological constructs, and health outcomes—are sufficiently consistent across countries to justify combining national samples in pooled analyses. Similarly, although the GFS includes rich self-reported data on physical and mental health, there has been little exploration of how these subjective indicators relate to objective measures such as healthcare utilization or mortality. As a result, the extent to which single-method bias may influence observed associations remains largely unknown. These are important areas for future methodological work to determine the validity and generalizability of findings derived from the GFS, especially in light of potential cultural differences in response styles and health perception. Our study should therefore be viewed as an early step toward identifying cross-national patterns, with the understanding that future research is needed to further validate the comparability and external validity of these measures across diverse global contexts.

### 5.1. Public Policy Implications

These findings offer several implications for public policy. If a sense of mastery and control over one’s life mediates the effects of both adversity and advantage on health, behaviors, and well-being, then policymakers should consider strategies that go beyond structural reforms alone and incorporate components that foster psychological empowerment. While improving access to education, employment, and financial support remains essential, these resources may be more effective when coupled with policies that promote agency, autonomy, and participation. For example, labor and welfare policies that give people a sense of choice, voice, and predictability—such as flexible working conditions [37], community-based development programs, and participatory budgeting—may strengthen individuals’ sense of control, thereby enhancing health outcomes. Additionally, programs targeting marginalized populations should explicitly acknowledge and address the disempowering effects of structural disadvantage, learned hopelessness, and trauma. Social policies that are trauma-informed and empowerment-oriented may thus have broader population health benefits than those solely focused on material needs [38].

### 5.2. Psychotherapy and Clinical Implications

From a clinical and psychotherapeutic perspective, our results underscore the importance of incorporating sense of mastery-building techniques into mental health and behavioral health interventions. Therapeutic approaches such as cognitive–behavioral therapy (CBT) [39], acceptance and commitment therapy (ACT) [40], and narrative therapy [41] already contain elements that seek to restore or build an individual’s sense of control over life circumstances. Clinicians may wish to more explicitly frame treatment goals around enhancing sense of mastery—especially in patients who have experienced early life adversity, financial insecurity, or ongoing systemic disadvantage. Restoring a sense of control could improve engagement, treatment adherence, and outcomes across multiple domains. Furthermore, sense of mastery-enhancing interventions [42,43] may be especially useful in prevention and early intervention settings, such as school-based or community mental health programs. Training clinicians to assess and support perceived control as part of routine practice may enhance the overall effectiveness of psychological care, particularly for populations most affected by social adversity.

### 5.3. Implications for Trauma-Informed Work

The results of this study have important implications for trauma-informed practice across healthcare, education, social services, and community programs [44,45,46]. Individuals who have experienced early life adversity, such as childhood abuse, are at risk not only for psychological distress but also for a diminished sense of mastery and control over their lives [47]—a mechanism that may, in turn, mediate long-term health, behavioral, and well-being outcomes [48,49]. Trauma-informed approaches emphasize safety, trustworthiness, empowerment, and choice, which directly align with the goal of restoring individuals’ sense of control. Interventions and systems grounded in trauma-informed principles should intentionally foster environments where individuals are supported in regaining agency and influence over their decisions, routines, and futures [50,51,52]. This includes ensuring that service delivery systems do not inadvertently reproduce disempowerment—through rigid bureaucracies, punitive policies, or lack of voice—but instead are structured to promote autonomy and meaningful participation. Programs that help trauma survivors develop goal-setting skills, recognize their own strengths, and engage in decision-making processes may be especially effective in enhancing sense of mastery and interrupting the cycle by which trauma undermines long-term health and behavioral outcomes [53]. Integrating sense of mastery as a core outcome and process within trauma-informed work may thus deepen its impact and broaden its relevance across diverse populations.

### 5.4. Implications for Behavioral Interventions

The identification of sense of mastery and control over one’s life as a key mediator linking social conditions to health, behaviors, and well-being also offers practical direction for the design of behavioral interventions. Traditionally, behavioral interventions focus on skill-building, habit formation, and risk reduction [54,55,56,57]; however, our findings suggest that incorporating components aimed at enhancing perceived control could substantially strengthen these approaches [58]. Because sense of mastery is a subjective and modifiable psychological resource, it can be deliberately targeted and cultivated through intervention curricula [59,60,61]. This could involve structured goal-setting exercises, self-monitoring strategies, decision-making skill development, and reflections on personal agency. Even brief behavioral interventions—such as those used for smoking cessation, physical activity promotion, or stress reduction—may benefit from modules that increase individuals’ confidence in their ability to shape their own outcomes [28,62,63,64]. Embedding sense of mastery-enhancing strategies into the design and delivery of behavioral programs could make them more effective and sustainable, particularly for individuals whose social circumstances have historically limited their sense of agency [29,65,66].

### 5.5. Health in All Policies and Cross-Sector Collaboration

The findings also support the relevance of a “Health in All Policies” (HiAP) framework [67,68,69], which emphasizes that population health is shaped by decisions across all sectors of society—not just within the healthcare system [70,71]. If perceived control is a key psychological mediator linking social conditions to health, then interventions to boost sense of mastery need not be confined to clinical settings. Sectors such as education, labor, housing, transportation, and banking can all contribute to environments that foster or erode an individual’s sense of control. For instance, employment policies that reduce precarious work and promote job security, financial systems that enhance access to credit without predatory practices, or education systems that promote student agency and engagement may all help cultivate a population with stronger control beliefs—and therefore better health, behaviors, and well-being. Public health practitioners and policymakers should work collaboratively across sectors to identify leverage points where systems can be redesigned not only to improve material conditions but also to support individuals’ ability to influence their own lives.

One challenge in interpreting our findings relates to cultural variation in the meaning and expression of perceived control or sense of mastery. While these psychological constructs are often considered protective in Western contexts, their interpretation and relevance may differ across cultural settings. For example, in more collectivist societies, individual control may be less emphasized or even discouraged in favor of interdependence and social harmony. As a result, the same level of sense of mastery may not carry equivalent psychological or behavioral implications across countries, which complicates both interpretation and cross-national comparisons. This cultural variability may partly explain some of the unexpected or difficult-to-justify findings in our study and suggests caution when generalizing results across diverse populations.

Importantly, the consistency of mediation effects in a cross-national sample composed of 23 countries in the Global Flourishing Study suggests that sense of control may operate as a relatively universal psychological mechanism. While cultural variation in the experience and expression of control is certainly possible—and should be explored in future work—the robustness of the observed patterns lends support to the generalizability of sense of mastery as a mediator of social conditions across global populations. Nonetheless, further research is needed to assess whether the strength of these pathways varies by cultural, economic, or policy contexts.

### 5.6. Limitations and Strengths

This study has several strengths. It draws on a large, diverse international sample with harmonized measures of key social, psychological, and outcome variables. It simultaneously models multiple social determinants and a broad range of outcomes, providing a comprehensive view of how sense of mastery may operate across domains. However, limitations should also be acknowledged. While in this study we used the terms sense of mastery, perceived control, and interchangeably, these constructs slightly differ and have differential correlates across diverse populations [72,73,74,75,76].

A key limitation of the current study is the use of single-item measures to assess complex constructs such as sense of mastery, health, and happiness. While these items are widely used in large-scale surveys due to their brevity and ease of administration, they may lack the depth and nuance required to fully capture multidimensional psychological and health-related experiences. Specifically, the use of a single item to measure mastery raises concerns about construct validity, as it may not reflect the broader domain of perceived control or agency. We acknowledge this limitation and recommend that future research employ validated multi-item scales to assess these constructs more comprehensively, which would enhance the reliability and interpretability of findings across diverse cultural and contextual settings.

The cross-sectional design does not allow for conclusions about causality or the temporal ordering of effects. All variables were self-reported, which may introduce measurement bias or reflect differences in reporting across cultures. All variables were self-reported, which raises concerns of common method variance. Additionally, we did not test for moderation by country, gender, or age, which may reveal important heterogeneity in these pathways.

### 5.7. Research Directions

Future research should build on these findings by using longitudinal data to test the stability and directionality of these effects over time. Subgroup analyses could help identify populations for whom sense of mastery is especially influential or vulnerable. Other potential mediators, such as optimism [77,78], social support, or religious engagement, could be tested in parallel to better understand the psychological landscape that links social structure to individual well-being. Moreover, efforts to culturally validate the sense of mastery measure and examine local interpretations of control could strengthen the cross-cultural relevance of this work.

## 6. Conclusions

In conclusion, this study suggests that sense of mastery and control over life is a robust psychological mediator that helps explain how both stressors and socioeconomic resources shape health, behaviors, and well-being. Mastery may serve as a bridge between the social world and individual functioning, making it a promising target for interventions aimed at reducing the negative effects of adversity and enhancing the benefits of social privilege. Strengthening individuals’ sense of control may be a vital pathway toward global health equity and human flourishing.

## Figures and Tables

**Table 1:** Number and percentage of participants across countries.

	n	%	SE	95%	CI
Argentina	6724	3.23	0.04	3.16	3.31
Australia	3844	1.85	0.03	1.79	1.91
Brazil	13,203	6.35	0.05	6.25	6.46
Egypt	4729	2.27	0.03	2.21	2.34
Germany	9506	4.57	0.05	4.48	4.66
India	12,765	6.14	0.05	6.04	6.24
Indonesia	6992	3.36	0.04	3.29	3.44
Israel	3669	1.76	0.03	1.71	1.82
Japan	20,543	9.88	0.07	9.75	10.01
Kenya	11,389	5.48	0.05	5.38	5.58
Mexico	5776	2.78	0.04	2.71	2.85
Nigeria	6827	3.28	0.04	3.21	3.36
Philippines	5292	2.55	0.03	2.48	2.61
Poland	10,389	5	0.05	4.90	5.09
South Africa	2651	1.28	0.02	1.23	1.32
Spain	6290	3.03	0.04	2.95	3.10
Tanzania	9075	4.36	0.04	4.28	4.45
Türkiye	1473	0.71	0.02	0.67	0.75
United Kingdom	5368	2.58	0.03	2.51	2.65
United States	38,312	18.43	0.09	18.26	18.59
Sweden	15,068	7.25	0.06	7.14	7.36
Hong Kong	3012	1.45	0.03	1.40	1.50
China	5022	2.42	0.03	2.35	2.48
Total	207,919	100			

**Table 2:** Summary of SEM on association between social determinants and health and well-being.

	B	SE	0.950	CI	*p*
Structural					

Sense of Mastery					
Educational Attainment	−0.006	0.002	−0.011	−0.002	0.004
Married	0.045	0.002	0.041	0.050	<0.001
Age (Year)	−0.058	0.002	−0.063	−0.054	<0.001
ACE (Abused as a Child)	−0.013	0.002	−0.017	−0.008	<0.001
Male	0.028	0.002	0.024	0.032	<0.001
Financial Insecurity	−0.152	0.002	−0.156	−0.147	<0.001
Unemployed	−0.007	0.002	−0.012	−0.003	0.001

Anxiety					
Sense of Mastery	−0.089	0.002	−0.093	−0.085	<0.001
Educational Attainment	−0.065	0.002	−0.070	−0.061	<0.001
Married	−0.040	0.002	−0.045	−0.036	<0.001
Age (Year)	−0.138	0.002	−0.143	−0.134	<0.001
ACE (Abused as a Child)	0.108	0.002	0.104	0.112	<0.001
Male	−0.044	0.002	−0.048	−0.040	<0.001
Financial Insecurity	0.224	0.002	0.220	0.229	<0.001
Unemployed	0.040	0.002	0.036	0.044	<0.001

Depression					
Sense of Mastery	−0.143	0.002	−0.147	−0.139	<0.001
Educational Attainment	−0.092	0.002	−0.096	−0.088	<0.001
Married	−0.050	0.002	−0.055	−0.046	<0.001
Age (Year)	−0.123	0.002	−0.128	−0.119	<0.001
ACE (Abused as a Child)	0.106	0.002	0.102	0.110	<0.001
Male	−0.022	0.002	−0.026	−0.018	<0.001
Financial Insecurity	0.216	0.002	0.211	0.220	<0.001
Unemployed	0.052	0.002	0.047	0.056	<0.001

Physical SRH					
Sense of Mastery	0.243	0.002	0.239	0.247	<0.001
Educational Attainment	0.000	0.002	−0.005	0.004	0.837
Married	0.055	0.002	0.051	0.060	<0.001
Age (Year)	−0.200	0.002	−0.205	−0.196	<0.001
ACE (Abused as a Child)	−0.074	0.002	−0.078	−0.070	<0.001
Male	−0.002	0.002	−0.006	0.002	0.251
Financial Insecurity	−0.213	0.002	−0.217	−0.208	<0.001
Unemployed	0.018	0.002	0.014	0.022	<0.001

Mental SRH					
Sense of Mastery	0.309	0.002	0.305	0.313	<0.001
Married	0.084	0.002	0.079	0.088	<0.001
Age (Year)	−0.002	0.002	−0.006	0.002	0.355
ACE (Abused as a Child)	−0.102	0.002	−0.106	−0.098	<0.001
Male	0.001	0.002	−0.003	0.005	0.708
Financial Insecurity	−0.191	0.002	−0.195	−0.187	<0.001
Unemployed	0.024	0.002	0.020	0.028	<0.001

Happiness					
Sense of Mastery	0.240	0.002	0.236	0.244	<0.001
Educational Attainment	0.028	0.002	0.024	0.032	<0.001
Married	0.075	0.002	0.071	0.079	<0.001
Age (Year)	0.004	0.002	−0.001	0.008	<0.001
ACE (Abused as a Child)	−0.052	0.002	−0.056	−0.048	<0.001
Male	−0.053	0.002	−0.057	−0.049	<0.001
Financial Insecurity	−0.284	0.002	−0.288	−0.280	<0.001
Unemployed	−0.060	0.002	−0.064	−0.056	<0.001

Life Satisfaction					
Sense of Mastery	0.175	0.002	0.171	0.179	<0.001
Educational Attainment	0.137	0.002	0.133	0.141	<0.001
Married	0.035	0.002	0.031	0.039	<0.001
Age (Year)	0.060	0.002	0.056	0.064	<0.001
ACE (Abused as a Child)	−0.019	0.002	−0.023	−0.015	<0.001
Male	−0.047	0.002	−0.051	−0.043	<0.001
Financial Insecurity	−0.288	0.002	−0.292	−0.284	<0.001
Unemployed	−0.051	0.002	−0.055	−0.047	<0.001

Exercise					
Sense of Mastery	0.085	0.002	0.081	0.090	<0.001
Educational Attainment	0.009	0.002	0.004	0.013	<0.001
Married	0.006	0.002	0.001	0.010	0.011
Age (Year)	0.003	0.002	−0.001	0.008	0.174
ACE (Abused as a Child)	0.009	0.002	0.004	0.013	<0.001
Male	0.083	0.002	0.078	0.087	<0.001
Financial Insecurity	−0.052	0.002	−0.056	−0.047	<0.001
Unemployed	−0.001	0.002	−0.005	0.003	0.666

Smoking					
Sense of Mastery	−0.012	0.002	−0.016	−0.007	<0.001
Educational Attainment	−0.056	0.002	−0.061	−0.052	<0.001
Married	−0.053	0.002	−0.057	−0.048	<0.001
Age (Year)	−0.004	0.002	−0.009	0.000	0.061
ACE (Abused as a Child)	0.039	0.002	0.035	0.044	<0.001
Male	0.159	0.002	0.155	0.164	<0.001
Financial Insecurity	0.056	0.002	0.051	0.060	<0.001
Unemployed	−0.005	0.002	−0.010	−0.001	0.018

Drinking					
Sense of Mastery	−0.017	0.002	−0.022	−0.013	<0.001
Educational Attainment	0.193	0.002	0.189	0.197	<0.001
Married	−0.040	0.002	−0.045	−0.036	<0.001
Age (Year)	0.111	0.002	0.107	0.116	<0.001
ACE (Abused as a Child)	0.024	0.002	0.020	0.028	<0.001
Male	0.151	0.002	0.146	0.155	<0.001
Financial Insecurity	−0.047	0.002	−0.052	−0.043	<0.001
Unemployed	−0.039	0.002	−0.043	−0.035	<0.001

## Data Availability

We used publicly available data from the Global Flourishing Study (GFS), which are accessible through the Center for Open Science at https://www.cos.io/gfs-access-data (accessed on 22 June 2025). The GFS data are openly available, and any interested individual can apply for access through the platform.
